# Synuclein gamma predicts poor clinical outcome in colon cancer with normal levels of carcinoembryonic antigen

**DOI:** 10.1186/1471-2407-10-359

**Published:** 2010-07-07

**Authors:** Caiyun Liu, Bin Dong, Aiping Lu, Like Qu, Xiaofang Xing, Lin Meng, Jian Wu, Y Eric Shi, Chengchao Shou

**Affiliations:** 1 Key laboratory of Carcinogenesis and Translational Research (Ministry of Education), Peking University School of Oncology, 52 Fucheng Road, Haidian District, Beijing 100142, China; 2 Department of Biochemistry & Molecular Biology, Peking University School of Oncology, Beijing Cancer Hospital & Institute, 52 Fucheng Road, Haidian District, Beijing 100142, China; 3 Department of Pathology, Peking University School of Oncology, Beijing Cancer Hospital & Institute, 52 Fucheng Road, Haidian District, Beijing 100142, China; 4 Department of Radiation Medicine, Long Island Jewish Medical Center, The Feinstein Institute for Medical Research, 270-05 76th Avenue, New Hyde Park, NY 11040, USA

## Abstract

**Background:**

Synuclein gamma (SNCG), initially identified as a breast cancer specific gene, is aberrantly expressed in many different malignant tumors but rarely expressed in matched nonneoplastic adjacent tissues. In this study, we investigated the prognostic potential of SNCG in colon cancer particularly in the patients with normal carcinoembryonic antigen (CEA) levels.

**Methods:**

SNCG levels were assessed immunohistochemically in cancer tissues from 229 colon adenocarcinoma patients with a mean follow-up of 44 months. Correlations between SNCG levels and clinicopathologic features, preoperative serum CEA level, and clinical outcome were analyzed statistically using SPSS.

**Results:**

SNCG levels in colon adenocarcinoma were closely associated with intravascular embolus and tumor recurrence but independent of preoperative serum CEA levels. SNCG expression was an independent prognostic factor of a shorter disease-free survival (DFS) and overall survival (OS) (*P *< 0.0001). Multivariate analysis revealed that both tissue SNCG and serum CEA were independent prognostic factors of DFS (*P *= 0.001, <0.0001, respectively) for 170 patients with colon adenocarcinomas. Importantly, SNCG remained a prognostic determinant of DFS and OS (*P *= 0.001, 0.002) for 97 patients with normal preoperative serum CEA level.

**Conclusions:**

Our results suggest for the first time that SNCG is a new independent predicator for poor prognosis in patients with colon adenocarcinoma, including those with normal CEA levels. Combination of CEA with SNCG improves prognostic evaluation for patients with colon adenocarcinoma.

## Background

Colorectal cancer (CRC) is one of the most common cancer types worldwide and it continues to be a serious public health problem. Traditionally, TNM stage is the most important predictor of survival for CRC patients, but current classification of CRC can't predict prognosis precisely even for the patients in the same clinical stage. Approximate 30% of stages I-II and 60% of stage III CRC patients develop recurrence in two years after surgery [1]. It is critical to find molecular signatures or factors for predicting prognosis and for selecting high-risk patients who need preventive chemotherapy or other adjuvant therapies [2]. CEA is a widely used tumor markers worldwide in CRC [3]. Serial monitoring of serum CEA for predicting recurrence and prognosis of CRC has been established [4]. However, lack of sensitivity and specificity preclude the use of CEA. Approximate 30% of all CRC recurrences do not have elevated CEA serum levels [5]. Since any single marker is not sufficiently predictive, combination of different markers representing different aspects of tumor biology will have a better prognostic evaluation [6,7]. Therefore, new cancer biomarkers or better surveillance methods should be developed, evaluated and standardized to improve the diagnostics of the disease [1,4].

Synucleins are a family of small proteins consisting of 3 known members, synuclein α (SNCA), synuclein β (SNCB), and SNCG [8]. While synucleins are highly expressed in neuronal cells and are abundant in presynaptic terminals, SNCA and SNCB have been specifically implicated in neurodegenerative diseases [9,10]. SNCG, initially identified as a breast cancer specific gene [11], is not clearly involved in neurodegenerative diseases but primarily involved in neoplastic diseases [11-16]. SNCG overexpression in breast cancer cells stimulates proliferation, induces metastasis, promotes chromosomal instability, inhibits mitotic checkpoint [12,17-19], and increases resistance to certain chemotherapeutic or anti-microtubule agents [20,21], however down-regulation of SNCG expression sensitizes breast cancer cells to anti-microtubule agents-induced cytotoxicity [20-22]. Being identified as a breast cancer specific gene, SNCG is aberrantly expressed in malignant breast cancer cells but not in the adjacent normal cells [16]. So far, the abnormal expression of SNCG protein has been demonstrated in 12 different malignant diseases, including ovarian [13,23], liver [15,24], esophagus [15], colon [15,25], gastric [15], lung [15], prostate [15], pancreas [26], bladder [27], cervical cancers [15], and glial tumors[28]. In these studies, SNCG protein is abnormally expressed in a high percentage of tumor tissues but rarely expressed in tumor-matched nonneoplastic adjacent tissues.

The clinical relevance of SNCG expression on breast cancer prognosis was confirmed in clinical follow-up studies [16,29]. Patients with an SNCG-positive tumor had a significantly shorter disease-free survival and overall survival compared with those with no SNCG expression. However, the prognostic significance of SNCG in other cancers remains unknown. In the current study, SNCG level as assessed by immunohistochemistry of tumor sections is an independent prognostic factor of a shorter DFS and OS for colon cancer patients. Importantly, SNCG remains a prognostic determinant of DFS and OS for colon cancer patients with normal preoperative serum CEA level.

## Methods

### Study Patients

Two hundred and twenty-nine colon adenocarcinoma tissue, 194 corresponding non-neoplastic adjacent tissue, and 37 colon adenoma, hyperplasia and polyp specimens were obtained from the archives (1996-2003) of the Department of Pathology, Peking University School of Oncology. Specimens from patients were diagnosed histopathologically and staged according to the TNM-International Union against Cancer classification system. The clinicopathologic characteristics of patients were described in Table 1. Among 229 colon cancer patients, 66 (28.8%) patients had tumor origin in the right colon, 16 (7%) patients had tumor origin in the transverse colon, and 147 (64.2%) patients had tumor origin in the left colon. None of the patients had received chemotherapy or radiation therapy before surgery, and none of them had a history of hereditary. Most primary tumors were treated by surgical resection and 20 patients including 17 stage IV patients received palliative treatment. Age at first diagnosis ranged from 23 to 85 years (mean ± SD, 60.6 ± 12.1). The mean follow-up length for these patients was 44.1 ± 28.2 months ranged from 11 days to 121 months. During follow-up, 46.3% (106/229) patients died of colon adenocarcinoma and 26.9% (61/229) patients developed recurrence after surgery. Among the patients with recurrence, liver metastasis was present in 28 of 61 (46%) cases, lung metastasis was present in 9 of 61 (15%), extensive intra-abdominal metastases in 11 of 61 (18%), and the rest recurrent lesions were in colon, brain, bone, and ovary. Overall survival time was calculated from the date of surgery to the date of death due to any cause. Disease-free survival (DFS) time was calculated for patients from the date of surgery to the date of disease progression (local recurrence or distant metastasis). Data on patients, who had survived until the end of follow-up period, were censored at the date of last contact. Informed consent was obtained from all of the patients and healthy examinees. The study was approved and supervised by the Medical Ethic Committee of Beijing Cancer Hospital/Institute.

**Table 1:** Correlations of SNCG Expression with Clinicopathologic Factors and Their Influences on Postoperative Recurrence

Characteristics	No. of cases	SNCG expression		No. of recurrence (%)	HR (95%CI)	*P *value
		SNCG+ (%)	*P *value			
Gender						
Male	121	38 (31.4)	0.066	28 (23.1)	1	0.205
Female	108	36 (33.3)		33 (30.6)	1.461 (0.811-2.632)	
Age (yrs)						
≤60	89	31 (34.8)	0.516	22 (24.7)	1	0.601
>60	140	43 (32.9)		39 (27.9)	1.176 (0.641-2.581)	
Size (cm)						
≤4	90	23 (25.6)	0.072	19 (21.1)	1	0.120
>4	138	51 (37.0)		42 (30.4)	1.635 (0.877-3.047)	
TNM stage						
I/II	114	32 (28.1)	0.172	14 (12.3)	1	0.000
III/IV	115	42 (36.5)		47 (40.9)	4.937 (2.522-9.663)	
Depth of invasion						
pT1 and pT2	39	12 (30.8)	0.821	4 (10.3)	1	0.011
pT3	190	62 (32.6)		57 (30.0)	3.750 (1.273-11.043)	
Differentiation						
WD and MD	192	61 (31.8)	0.689	49 (25.5)	1	0.384
PD	37	13 (35.1)		12 (32.4)	1.401 (0.654-2.998)	
LN metastasis						
Negative	125	37 (29.6)	0.336	20 (16.0)	1	0.000
Positive	104	37 (35.6)		41 (39.4)	3.417 (1.840-6.346)	
Intravascular embolus						
Negative	170	48 (28.2)	0.025	40 (23.5)	1	0.071
Positive	59	26 (44.1)		21 (35.6)	1.796 (0.947-3.406)	
CEA						
Negative	97	34 (35.1)	0.658	15 (15.5)	1	0.001
Positive	73	28 (38.4)		28 (38.4)	3.401 (1.648-7.023)	
ND	59					
SNCG						
Negative	155	----	----	34 (22.2)	1	0.020
Positive	74	----		27 (36.5)	2.044 (1.114-3.752)	

WD, well-differentiated; MD, moderately differentiated; PD, poorly differentiated; LN, lymph node; HR, hazard ratio; CI, confidence interval; ND, Not Detected.

### Immunohistochemistry

SNCG protein expression was analyzed by immunohistochemical staining as the following procedures. Paraffin-embedded whole tissue sections were deparaffinized with xylene. Following rehydration in distilled water, antigen was retrieved by heating in EDTA (pH 8.0, Zymed). Endogenous peroxidase activity was blocked by incubating in 3% hydrogen peroxide at room temperature (RT) for 10 minutes. Nonspecific binding was blocked with PBST (0.01 mol/L PBS containing 0.05% Tween-20) containing 10% goat serum and 3% skimmed milk for 2 h at RT. Anti-SNCG mAb 1# [25] was applied to each slide and incubated at RT for 2 h. Following three washes, slides were incubated with Envision (DAKO) for 40 minutes at RT. Diaminobenzidine was used as a chromogen. Sections were counterstained with hematoxylin, dehydrated, and mounted. The quality, specificity, and sensitivity of the assay were determined in reference 25. As a positive control, a colon cancer tissue with confirmed strong and SNCG specific staining in previous study [25] was used, whereas the primary antibody was omitted for a negative control.

### Evaluation of Immunohistochemical Staining

Immunohistochemical expression was evaluated under light microscopy (APPLIED IMAGING at 200×) independently by two experienced pathologists (B Dong and A Lu) without knowledge of the patients' backgrounds and clinicopathologic data. There were 9 cases (3.9%) disagreement on weak staining and the discrepancies were resolved by simultaneous reevaluation. Immunoreactivity for SNCG in tumour cells was graded as either negative or positive according to a four-value classification scale as follows: area of staining as <10 percent (0) or >10 percent (1) of all cancer cells stained within the section, staining intensity (>10% of all cancer cells stained within the section) was graded as weak (1), moderate (2), or strong (3). A total score for area adding grade of 3 or more was defined as positive expression and less than 3 as negative. To avoid inappropriate evaluation caused by variations of background staining, all stained slides were reconciled with negative control slides from the same tissue samples.

### Preoperative CEA Value Determination

The preoperative serum levels of CEA were determined by commercially available immunoassay ELISA kit (Roche Diagnostics). The serum levels of CEA were considered positive when they were equal to or higher than 5.0 ng/ml (cutoff value) and negative when lower than that according to the manufacture's instructions.

### Data Analysis

Differences in SNCG protein expression between cancer and non-cancer tissues in the same patient were analyzed using a paired T test. Correlations between SNCG levels and patient clinicopathologic characteristics, CEA levels were performed using Pearson chi-square test. The Kaplan-Meier method was used to estimate disease-free survival (DFS) and overall survival (OS) rates, and the survival differences were analyzed by Log rank test. The Cox proportional hazard model was used for multivariate analysis to investigate the independence of the risk factors identified as significant in the univariate analysis. All statistical analyses were two-sided, and comparisons made in which probability values less than 0.05 were considered statistically significant. All statistical analyses were carried out using SPSS for Windows Software (version 13.0).

## Results

### SNCG is overexpressed in colon adenocarcinoma cells and is associated with intravascular embolus

**Figure 1:**
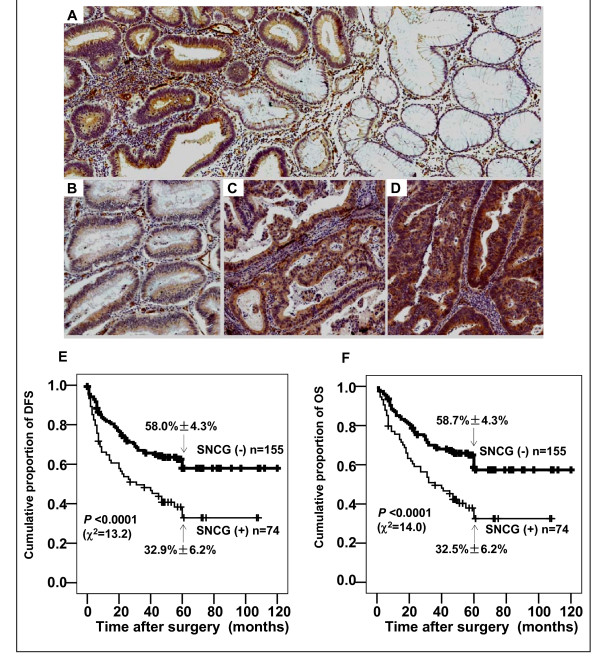
**Representative immunohistochemical staining for SNCG protein in human colon adenocarcinoma tissues (A-D) and Kaplan-Meier estimation of disease-free survival (E) and overall survival (F)**. A, SNCG expressed in the cytoplasm of colon cancer cells but not in the adjacent normal adenoepithelium. SNCG also expressed in colon neuron-chords, vascular endothelial cells, and smooth muscle cells of nearly all colon cancer specimens; B, C, and D represented the intensity of weak (1 score), moderate (2 scores) and strong (3 scores) staining of SNCG in cancer cytoplasm (original magnification 200×); E-F, Cases with SNCG-negative tumors (thick line) versus cases with SNCG-positive (thin line). SNCG-positive patients showed significantly poorer survival rates than those of SNCG-negative patients (*P *< 0.0001 by Log Rank (Mantel Cox) test). Numbers in the graph indicate percentages of DFS and OS at 5 year, respectively.

Using a previously characterized specific monoclonal antibody for SNCG [25], we immunohistochemically analyzed SNCG expression in 460 clinical colon samples including 37 benign adenoma, hyperplasia, and polyp tissues, 229 colon adenocarcinomas, and 194 tumor-adjacent normal epithelium. As summarized in Table 2, none of 37 benign lesions showed positive staining of SNCG. In contrast, SNCG was aberrantly expressed in colon adenocarcinomas. SNCG expression in colon adenocarcinoma was heterogeneous and varied greatly between different cancer cells. As shown in Figure 1A, SNCG specifically expressed in the cytoplasm of cancer cells, whereas no expression observed in the adjacent normal epithelium. Figure 1B, C, D represented the intensity of weak (1 score), moderate (2 scores) and strong (3 scores) staining of SNCG in cancer cells. We also found that SNCG was strongly expressed in colon neuron-chords, vascular endothelial cells, and smooth muscle cells of nearly all colon cancer specimens. While only 4 SNCG-positive cases were detected in 194 tumor-adjacent normal tissues (2.1%), moderate or strong expression of SNCG protein was detectable in 74 of 229 colon cancer cases (32.3%).

**Table 2:** SNCG Expressing Profile in Colon Adenocarcinoma

	cases	SNCG expression		Positive rate (%)	*P *value
		(-)	(+)		
Normal^a^	194	190	4	2.1	
Benign^b^	37	37	0	0	
Colon adenocarcinoma tissues (n = 229)					
Stage I	31	21	10	32.3	0.001#
Stage II	83	61	22	26.5	<0.0001#
Stage III	66	48	18	27.3	<0.0001#
Stage IV	49	25	24	49.0	<0.0001#
Total(I-IV)	229	155	74	32.3	<0.0001#

^a^tumor-adjacent normal epithelium; ^b^Adenoma, hyperplasia, and polyp tissues; ^#^difference between adenocarcinoma tissues *versus *tumor-adjacent normal epithelium tissues (paired T test).

Associations between SNCG expression and clinical and biological tumor characteristics were analyzed (Table 1). Overall, there was no significant correlation between SNCG protein expression and age, tumor size, tumor differentiation, depth of invasion, TNM stage, and preoperative serum CEA levels. However, expression of SNCG in colon adenocarcinoma tissues was strongly correlated with intravascular embolus (*P *= 0.025). Interestingly, in contrast to previous observations of an association between SNCG expression and tumor stage in many different cancers [11,15], levels of SNCG in colon adenocarcinoma tissues did not display any significant difference between stages I-II and III-IV [28.1% (32/114) and 36.5% (42/115), *P *= 0.172]. The associations between these factors and recurrence were also analyzed (Table 1). As expected, clinicopathologic features including TNM stage (*P *< 0.0001), lymph node metastasis (*P *< 0.0001), depth of invasion (*P *= 0.011), preoperative serum CEA levels (*P *= 0.001) significantly influenced recurrence of colon adenocarcinoma, whereas intravascular embolus, histological differentiation, gender, age, and tumor size didn't affect recurrence of tumors (*P *> 0.05). Expression of SNCG in primary tumors (*P *= 0.023) was also significantly associated with recurrence. There were 61 patients developed tumor recurrence during the follow-up period. While 37% of patients with SNCG positive primary tumor developed tumor recurrence, only 22% of patients with SNCG negative tumors developed tumor recurrence (*P *= 0.02).

### SNCG overexpression correlates with poor outcome and is an independent prognostic indicator

To study whether SNCG is a prognostic factor for colon cancer, we correlated SNCG expression in tumors with a median follow-up of 44 ± 28 months (range 11 days to 121 months) after colon cancer surgery. We found a strong association between SNCG and survival. SNCG-positive patients showed a significantly poorer prognosis than SNCG-negative patients in Kaplan-Meier analysis of disease free survival (DFS) and overall survival (OS) (Figure 1E, F). While the cumulative proportions of DFS and OS at 5 year after surgery were 58.0% ± 4.3%, 58.7% ± 4.4% in the SNCG-negative group, for the SNCG-positive group, those of DFS and OS were reduced to 32.9% ± 6.2% and 32.5% ± 6.2%, respectively. The mean time for DFS and OS was 78.7 ± 4.2 months [95% confidence interval (CI), 70.5-86.9 months] and 80.3 ± 4.0 months (95%CI, 72.4-88.2 months) in the SNCG negative group, 48.7 ± 5.4 months (95%CI, 38.1-59.3 months) and 51.1 ± 5.2 months (95%CI, 40.9-61.2 months) in the SNCG positive group (*P *< 0.0001, respectively). We also found SNCG levels were positively correlated with recurrence (*r *= 0.141, *P *= 0.020) and inversely correlated with survival (*r *= -0.221, *P *= 0.001, Spearman's correlation coefficient) of patients with colon adenocarcinoma. The hazard ratio of recurrence and death according to SNCG level was 2.044 (95% CI, 1.114-3.752, *P *= 0.020) and 2.601 (95%CI, 1.471-4.601, *P *= 0.001).

Multivariate analysis revealed that SNCG was an independent prognostic factor for DFS (*P *= 0.039) and OS (*P *= 0.048) of the patients with colon adenocarcinoma. SNCG level in colon adenocarcinoma tissue was predictive for development of recurrence and a shorter DFS/OS.

### Combination of SNCG and CEA improves prognostic value of patients with colon adenocarcinoma

Since SNCG level was not associated with preoperative serum CEA level, we were interested in studying whether a combination of SNCG and CEA could improve prognostic evaluation. As illustrated in Table 3, multivariate analyses indicate that CEA, SNCG, and combination of CEA and SNCG all remained independent prognostic factors for DFS (*P *< 0.0001, 0.001, <0.0001) and OS (*P *< 0.0001, 0.001, <0.0001, respectively). Very importantly, the hazard ratio of combined CEA and SNCG for DFS and OS were 3.517 and 3.645, 2.440 and 2.639 for CEA, and 2.213 and 2.141 for SNCG, respectively. These data suggested that the combination of CEA and SNCG was a strong prognostic indicator.

**Table 3:** Prognostic Value of Clinicopathological Factors, CEA, SNCG, and Combined CEA and SNCG on DFS and OS of 170 Patients with Colon Adenocarcinoma

Characteristics	DFS		OS	
	HR (95% CI)	*P *value	HR (95% CI)	*P *value
**Univariate analysis**				
TNM stage (III/IV vs. I/II)	4.745 (2.873-7.837)	<0.0001	4.667 (2.827-7.704)	<0.0001
Depth of invasion(pT3 vs. pT1, T2)	3.539 (1.489-8.410)	0.004	3.507 (1.476-8.331)	0.004
Differentiation (PD *vs. *WD, MD)	1.733 (1.130-2.658)	0.012	1.741 (1.144-2.649)	0.010
LN metastasis(Positive vs. Negative)	3.701 (2.303-5.946)	<0.0001	3.593 (2.237-5.771)	<0.0001
Intravascular embolus(Positive vs. Negative)	1.933 (1.202-3.106)	0.006	2.021 (1.257-3.248)	0.004
CEA (Positive vs. Negative)	2.761 (1.739-4.383)	<0.0001	2.776 (1.750-4.405)	<0.0001
SNCG (Positive vs. Negative)	2.146 (1.367-3.368)	0.001	2.121 (1.351-3.331)	0.001
SNCG/CEA (either positive vs. both negative)	3.756 (2.097-6.729)	<0.0001	3.811 (2.127-6.830)	<0.0001
**Multivariate analysis**				
TNM stage (III/IV vs. I/II)	3.251 (1.209-8.748)	0.020	3.904 (1.447-10.536)	0.007
Depth of invasion (pT3 vs. pT1, T2)	3.701 (1.449-9.450)	0.006	3.819 (1.488-9.805)	0.005
Differentiation (PD vs. WD, MD)	2.116 (1.309-3.422)	0.002	2.075 (1.313-3.299)	0.002
CEA (Positive vs. Negative)	2.440 (1.493-3.987)	<0.0001	2.639 (1.615-4.315)	<0.0001
SNCG (Positive vs. Negative)	2.213 (1.391-3.520)	0.001	2.141 (1.349-3.401)	0.001
SNCG/CEA (either positive vs. both negative)	3.517 (1.936-6.389)	<0.0001	3.645 (2.005-6.629)	<0.0001

WD, well-differentiated; MD, moderately differentiated; PD, poorly differentiated; LN, lymph node; HR, hazard ratio; CI, confidence interval.

**Figure 2:**
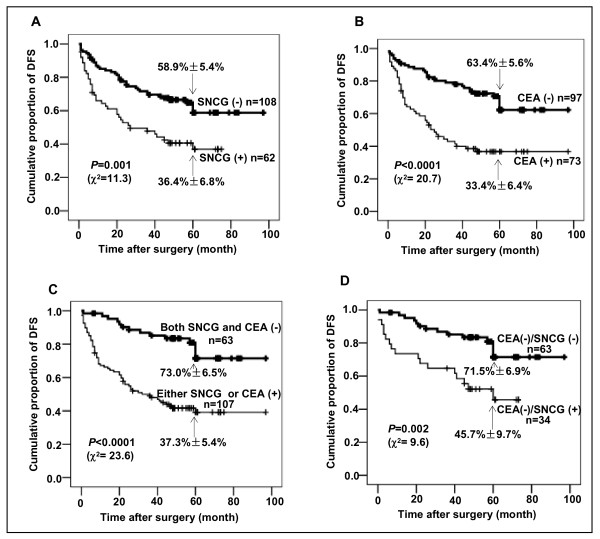
**Kaplan-Meier estimation of disease-free survival (DFS) for colon adenocarcinoma patients**. A-C, DFS in patients (n = 170) according to SNCG level, CEA level, and combined CEA with SNCG. Patients with SNCG positive (A), CEA positive (B), either SNCG or CEA positive (C) all showed significantly poorer DFS rates than those with the corresponding negative factors (*P *= 0.001, <0.0001, <0.0001, respectively by Log Rank (Mantel Cox) test). D, DFS in 97 patients with normal preoperative serum CEA level. SNCG-positive patients had a significantly poorer survival rate than those SNCG-negative patients (*P *= 0.002 by Log Rank (Mantel Cox) test). Numbers in the graph indicate percentages of DFS at 5 year, respectively.

Figure 2 illustrates that 170 patients with SNCG positive (A), CEA positive (B), and either SNCG or CEA positive (C) all show significantly poorer survival rates than those with the corresponding negative markers (*P *= 0.001, <0.0001, <0.0001, respectively). A significant difference in survival rate was observed in 5-year follow up. There were 59%, 63%, and 73% of DFS rates in patients with SNCG-negative, CEA-negative, and both SNCG- and CEA-negative, whereas SNCG-positive, CEA-positive, and either SNCG- or CEA-positive patients were 36%, 33%, and 37% (*P *= 0.001, <0.0001, <0.0001, respectively). During the follow-up period, 43 of 170 (25%) colon cancer patients were identified with postoperative relapse. While 39% (24/62) patients with SNCG-positive, 38% (28/73) CEA-positive, and 34% (36/107) either SNCG- or CEA-positive developed recurrence, only 18% (19/108) patients with SNCG-negative, 16% (15/97) CEA-negative, and 11% (7/63) both SNCG- and CEA-negative patients developed postoperative relapse (*P *= 0.002, 0.001, 0.001, respectively). The hazard ratio of recurrence according to combined SNCG with CEA was 4.056 (95%CI, 1.679-9.800, *P *= 0.001), 2.958 for SNCG (95% CI, 1.452-6.028, *P *= 0.002) and 3.401 for CEA (95%CI, 1.648-7.023, *P *= 0.001). Combination of CEA with SNCG might improve prognostic evaluation for patients with colon adenocarcinoma.

### SNCG overexpression correlates with poor outcome and remains an independent prognostic indicator for patients with normal preoperative serum CEA level

SNCG was overexpressed in 35% (34/97) of adenocarcinoma tissues from patients with normal preoperative serum CEA level. SNCG-positive patients showed a significantly poorer DFS rate than those SNCG-negative patients (*P *= 0.002, Figure 2D). The DFS rate at 5 year after surgery was 72% in the SNCG-negative group, and 46% in the SNCG-positive group. There were 42% (45/108) of patients with elevated CEA levels in the SNCG negative group, rates of DFS and OS of these patients were 43% and 39% at 5 year after surgery. Univariate analyses indicated that TNM stage (*P *< 0.0001), lymph node metastasis (*P *< 0.0001), intravascular embolus (*P *= 0.013), and depth of invasion (*P *= 0.044) significantly impacted the DFS and OS of these patients. However, in multivariate analysis, these factors were not correlative with DFS and OS of the patients with normal preoperative serum CEA level. In contrast, multivariate analysis indicated that SNCG level was the most important independent prognostic factor for DFS and OS (*P *= 0.001, 0.002), followed by tumor size (*P *= 0.020, 0.031) and differentiation grade (*P *= 0.023, 0.038). The hazard ratio of SNCG to DFS and OS were 3.491 and 3.132, while 2.734 and 2.545 for tumor size, and 2.372 and 2.035 for differentiation (Table 4). The data showed that tissue SNCG level was significantly correlated with patient clinical outcome and independent of other clinicopathological parameters for colon adenocarcinoma patients with normal preoperative serum CEA level.

**Table 4:** Prognostic Value of Clinicopathological Factors and SNCG on DFS and OS of 97 Patients with Normal CEA Level

Characteristics	DFS		OS	
	HR (95% CI)	*P *value	HR (95% CI)	*P *value
**Univariate analysis**				
TNM stage (III/IV vs. I/II)	5.703 (2.670-12.182)	<0.0001	5.298 (2.483-11.302)	<0.0001
Depth of invasion(pT3 vs. pT1, T2)	3.125 (1.030-9.478)	0.044	3.018 (0.997-9.132)	0.051
Differentiation (PD vs. WD, MD)	2.167 (1.089-4.312)	0.028	2.191 (1.107-4.338)	0.024
LN metastasis(Positive vs. Negative)	5.262 (2.466-11.224)	<0.0001	4.917 (2.307-10.480)	<0.0001
Intravascular embolus(Positive vs. Negative)	2.503 (1.214-5.161)	0.013	2.638 (1.279-5.418)	0.009
Tumor size (>4 cm vs. ≤4)	2.876 (1.237-6.683)	0.014	2.886 (1.242-6.705)	0.014
SNCG (Positive vs. Negative)	2.904 (1.428-5.905)	0.003	2.968 (1.458-6.044)	0.003
**Multivariate analysis**				
Differentiation (PD vs. WD, MD)	2.372 (1.128-4.990)	0.023	2.035 (1.042-3.977)	0.038
Tumor size (>4 cm vs. ≤4)	2.734 (1.169-6.394)	0.020	2.545 (1.089-5.951)	0.031
SNCG (Positive vs. Negative)	3.491 (1.656-7.359)	0.001	3.132 (1.506-6.511)	0.002

WD, well-differentiated; MD, moderately differentiated; PD, poorly differentiated; LN, lymph node; HR, hazard ratio; CI, confidence interval.

## Discussion

In the present study, we demonstrated that SNCG is an independent prognostic factor of a shorter survival for patients with colon adenocarcinoma. Although preoperative serum CEA levels may provide independent prognostic information [30], few studies have investigated the surveillance of patients with normal preoperative serum CEA levels. We investigated the impact of SNCG level on the clinical outcome of patients with normal preoperative serum CEA levels and our results demonstrated that SNCG remained an independent prognostic variable for these patients and affected patients' survival, but the clinicopathologic factors such as TNM stage, lymph node metastasis, depth of invasion, all didn't influence the patients' survival. Therefore, SNCG detection may represent a new prognostic tool for predicting relapse and survival outcome for patients with colon adenocarcinoma and particularly for the patients with normal preoperative serum CEA levels. We also demonstrated that combination of CEA and SNCG has a significant additive value and provides a high prognostic value in colon cancer. Tumor SNCG and preoperative CEA may provide mutual complementary prognostic value and combined analyses of SNCG with CEA provide a strong prognosis on survival outcome for patients with colon cancer.

SNCG levels in colon adenocarcinoma tissues are well correlated with the presence of intravascular embolus, but the impacts of SNCG on recurrence of tumor and on DFS/OS of patients are greatly stronger than intravascular embolus. Venous invasion or lymph node metastasis are generally recognized as prognostic clinicopathologic variables for hematogenic recurrence, which is the most frequent type of recurrence after surgery for CRC [31]. SNCG level in colon adenocarcinoma tissues may play a major role in hematogenous metastasis. Previously, we demonstrated that expression of SNCG in breast cancer cells leads to a significant increase in motility and a profound augmentation of metastasis in tumor xenograft [12]. In addition, we recently demonstrated that patients with SNCG-positive breast cancer have statistically higher incidence for metastasis compared with patients with SNCG-negative cancer [16]. It is anticipated that SNCG-stimulated cell motility and metastasis is mediated at least by its chaperoning activity on stimulation of activated (GTP-bound) form of Rho family members [32].

Previous studies indicate that SNCG expression follows a stage specific in breast cancer. While 71.4% of advanced breast cancers are positive for SNCG expression, only 26.8% of stage I/II breast cancers are positive for SNCG expression and 5.2% of benign hyperplasia expresses SNCG. SNCG protein is not detectable in normal tissue adjacent to breast cancer [16]. Similar studies also demonstrated that SNCG expression was stage-specific in many different cancer types [15]. However, in this study, we did not find any correlation between SNCG level and TNM stage. Relationship between SNCG level and TNM stage needs a further investigation. Interestingly, although our results revealed that elevated preoperative serum CEA level had a better prognostic value for the patients with stages I-II than the corresponding tissue SNCG level, SNCG levels predict the poor clinical outcome better than CEA level for patients with stages III-IV. These studies clearly demonstrated that SNCG may be useful as a prognostic indicator, especially important for patients with stages III-IV.

Tissue-based markers have been investigated for potential prognostic and predictive value. The most widely studied tissue markers in CRC are thymidylate synthase, microsatellite instability, p53, K-ras and deleted in colorectal cancer (DCC), but they have not currently been recommended in routine practice for determining prognosis or predicting response to therapy [4]. More accurate screening tests for CRC should be developed, including enhancing sensitivity of existing tumor markers and identifying new prognostic markers. Our immunohistochemical results showed that SNCG predominantly expressed in cytoplasm of colon cancer cells, but rarely in adjacent normal epithelium, which are consistent with previous report [25]. For some cancer cells, positive SNCG staining was also observed in nucleus and membrane, suggesting that SNCG is not an exclusively cytoplasmic protein. It has been previously reported that SNCG localizes to spindle poles [33] and translocates from perinuclear area to nucleus [34]. We also found that SNCG was highly expressed in colon neuron-chords, vascular endothelial cells and smooth muscle cells, but the biochemical and cellular function is still unknown.

## Conclusions

Our study demonstrated for the first time that tissue SNCG was an important prognostic indicator of shorter DFS/OS for CRC patients, especially for those with normal preoperative serum CEA level. SNCG level in colon adenocarcinoma is potentially valuable in predicting colon adenocarcinoma patients at high risk of recurrence and shorter survival after surgery. Tumor SNCG and preoperative CEA levels are mutually complementary prognostic factors and their combination improves prognostic evaluation of colon adenocarcinoma patients compared with each molecular marker alone. Interestingly, although SNCG gene does not have a signal peptide, suggesting it is not a secreted protein, a secreted form SNCG can be detected in serum [25,26] and urine [27] samples of malignant tumors. The potential application of serum levels of SNCG for diagnosis and prognosis of colon adenocarcinoma warrants further investigation.

## Competing interests

The authors declare that they have no competing interests.

## Authors' contributions

CL carried out the immunoassays, performed the statistical analysis and wrote the manuscript. BD participated in evaluation of immunohistochemical staining and histopathological interpretation. AL participated in evaluation of immunohistochemical staining. LQ helped to draft the manuscript and critically revised the manuscript. XX provided clinical samples and clinicopathological data. LM and JW participated in coordination of the study. YES participated in design, drafting of the manuscript and critically revised the manuscript. CS carried out the study conception, design, and drafting of the manuscript and critically revised the manuscript. All authors read and approved the final manuscript.

## Pre-publication history

The pre-publication history for this paper can be accessed here:

http://www.biomedcentral.com/1471-2407/10/359/prepub
